# Increased Diagnostic Certainty of Periprosthetic Joint Infections by Combining Microbiological Results with Histopathological Samples Gained via a Minimally Invasive Punching Technique

**DOI:** 10.3390/jcm9103364

**Published:** 2020-10-20

**Authors:** Andreas Enz, Johanna Becker, Philipp Warnke, Friedrich Prall, Christoph Lutter, Wolfram Mittelmeier, Annett Klinder

**Affiliations:** 1 Orthopädische Klinik und Poliklinik, Universitätsmedizin Rostock, Doberaner Straße 142, 18057 Rostock, Germany; johanna.becker@uni-rostock.de (J.B.); christoph.lutter@med.uni-rostock.de (C.L.); wolfram.mittelmeier@med.uni-rostock.de (W.M.); annett.klinder@med.uni-rostock.de (A.K.); 2 Institut für Medizinische Mikrobiologie, Virologie und Hygiene, Universitätsmedizin Rostock, Schillingallee 70, 18057 Rostock, Germany; philippchristoph.warnke@med.uni-rostock.de; 3 Institut für Pathologie, Universitätsmedizin Rostock, Strempelstraße 14, 18057 Rostock, Germany; friedrich.prall@med.uni-rostock.de

**Keywords:** biopsy, low grade, neosynovium, periprosthetic membrane, revision arthroplasty

## Abstract

Background: The diagnosis of low-grade infections of endoprostheses is challenging. There are still no unified guidelines for standardised diagnostic approaches, recommendations are categorised into major and minor criteria. Additional histopathological samples might sustain the diagnosis. However, ambulatory preoperative biopsy collection is not widespread. Method: 102 patients with hip or knee endoprosthesis and suspected periprosthetic joint infection (PJI) were examined by arthrocentesis with microbiological sample and histopathological punch biopsy. The data were retrospectively analysed for diagnosis concordance. Results: Preoperative microbiology compared to intraoperative results was positive in 51.9% (sensitivity 51.9%, specificity 97.3%). In comparison of preoperative biopsy to intraoperative diagnostic results 51.9% cases were positive (sensitivity 51.9%, specificity 100.0%). The combination of preoperative biopsy and microbiology in comparison to intraoperative results was positive in 70.4% of the cases (sensitivity 70.4%, specificity 97.3%). Conclusion: The diagnosis of PJI is complex. One single method to reliably detect an infection is currently not available. With the present method histopathological samples might be obtained quickly, easily and safely for the preoperative detection of PJI. A combination of microbiological and histopathological sampling increases the sensitivity up to 18.5% to detect periprosthetic infection.

## 1. Introduction

Periprosthetic joint infection (PJI) is one of the most challenging complications in patients with endoprosthetic treatment [1]. Beside aseptic loosening, it is the most common reason for revision surgery [2]. While the clinical diagnosis of a fulminant infection is quite obvious, the clinical discrimination of chronic infections (low-grade infections) from aseptic loosening is difficult but crucial in terms of choosing the correct treatment procedure [3,4]. While standard treatment methods are described in detail in the literature [4,5], no gold standard in diagnosis has yet been established. Also, a standard definition of periprosthetic infection has not yet been found. At the second International Consensus Meeting (ICM) on orthopaedic infection 2018 of the international consensus group in Philadelphia, an attempt was made to publish uniform diagnostic criteria, which largely correspond to the recommendations of the Musculoskeletal Infection Society (MSIS), but still show differences in detail. Two positive cultures from the joint with the same bacterial species and the formation of a sinus tract with communication to the affected joint are considered as major criteria. Minor criteria are serum and synovial markers, whereby in ICM a histopathological sample is included in the minor criteria, but in a preoperative setting the obtaining of a histopathological specimen and its information gain is underrepresented [6].

The arthrocentesis is one of the standard diagnostic procedures. In addition to microbiological cultural bound analyses, the synovial fluid can be examined for cell count or synovial fluid biomarkers [7] and microbiological multiplex polymerase chain reaction (PCR) approaches. The latter technique allows for rapid pathogen detection, also in combination with the detection of selected molecular antibiotic resistance markers [8,9]. In addition to microbiological analyses, the diagnosis of an infection can be fostered by histopathological examinations from parts of periprosthetic membranes as mentioned before [10,11]. According to the Morawietz classification for periprosthetic membranes, a higher diagnostic certainty for low-grade infections can be achieved [12,13]. Especially in the absence of common clinical signs, a problem that often occurs in low-grade infection, it is difficult to differentiate between aseptic and septic inflammation. Thus, it is as important to exclude the non-septic cases as it is to correctly diagnose PJI as this impacts on the patients’ treatment. So far, the sampling of the membrane in the pre-diagnosis of the infection has mostly been performed by arthroscopic surgery, with all the associated risks for the patients [14]. While the high value of histopathological sampling for the diagnosis of PJI was already proven for in-house patients [11], sample collection has always required a conventional surgical procedure. The novelty in this study was the implementation of a fast, safe, and easy-to-use, minimally invasive punching system for obtaining a synovial sample that works well in an outpatient setting under local anaesthesia. The collected samples were then evaluated with regard to increasing the reliability of diagnosis in PJI.

## 2. Experimental Section

### 2.1. Patients

Patients who had undergone arthrocentesis to obtain microbiological and histopathological samples at the Orthopaedic Clinic and Policlinic of the University Rostock Medical Center in the period from 01.04.2014–31.12.2017 to detect periprosthetic joint infections, were included in the study and retrospectively analysed. Inclusion criteria: all patients were over 18 years of age, able to give consent, had undergone primary or revision arthroplasty of hip or knee joint, showed signs of aseptic or septic loosening or periprosthetic joint infection and underwent subsequent revision surgery with intraoperative specimens taken for microbiological and histopathological testing. The decision to obtain microbiological and histopathological samples pre-operatively was based on medical history, history of infection or PJI, non-treatable pain, blood and inflammation parameters, the existence of fistula persistens, as well as certain radiological findings.

### 2.2. Technique and Biopsy System

**Figure 1:**
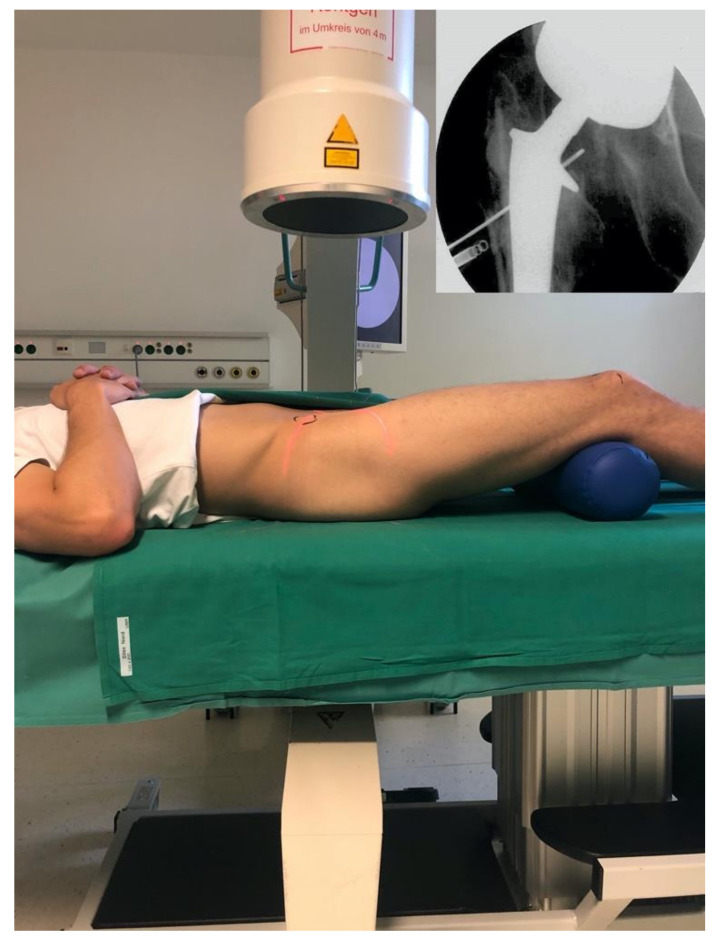
Position the patient for puncture of the hip joint. Spina iliaca anterior superior and middle of the patella are marked in black as corner points of the safe zone. Hip in 20° flexion.

The arthrocentesis of the hip joint was performed according to the in-house standard for hip arthrocentesis under strictly aseptic conditions (outpatient setup). The patient was positioned in a supine position with a round cushion under the hollow of the knee, which creates a flexion in the hip joint of about 20° (Figure 1). In this position, the posterior part of the hip joint capsule is tensed and ventrally relaxed, which simplifies puncture and punch biopsies [15,16]. The safe zone was subsequently marked. The safe zone is defined as the line from the spina iliaca anterior superior to the upper pole of the patella. The lateral area of this line is declared to be safe to the vascular nerve bundle. After thorough disinfection and localizing the arthrocentesis spot under fluoroscopic control, a skin paddle with local anaesthetic was placed subcutaneously, avoiding contact to the joint. In order to avoid collecting outer skin—thus transferring it including its skin microbiota and risking a potential infection of the joint—in the puncture cylinder, a skin incision (3 mm) was made in the paddle and the joint was punctured through the skin incision. In addition to the standard arthrocentesis of synovial fluid for microbiology, a sample was gained from the ventral capsule area via the same approach using an automatic biopsy system as further described below. The steps were controlled by fluoroscopy.

For the arthrocentesis and sampling of the knee joint, according to the in-house standard for knee arthrocentesis, the sample was gained via an approach of the lateral upper recess or via the lateral soft spot, also by means of a previous paddle incision. The sample of the capsule was taken from the ventral or anterolateral part of the capsule using the punch. In case of a dry tap during arthrocentesis in hip or knee joint, a second biopsy specimen was taken and microbiologically evaluated.

**Figure 2:**
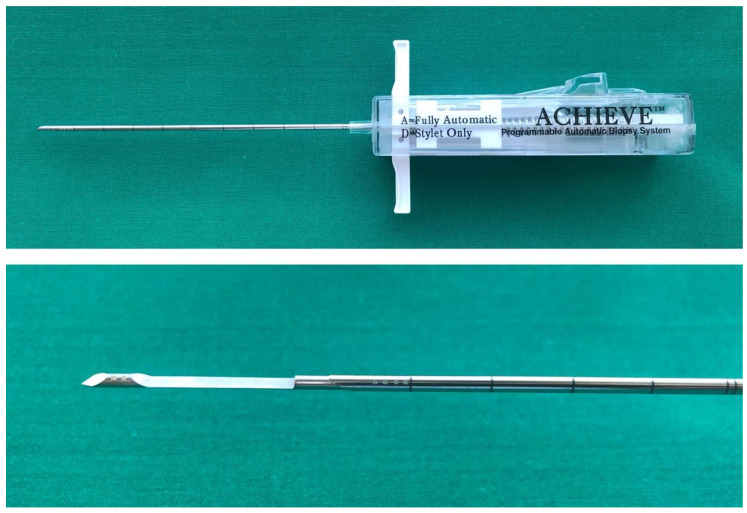
Programmable Automatic Biopsy System (Coaxial Achieve^®^, CareFusion Corporation, San Diego, CA, USA).

The used punching system for biopsy was a 14 G × 11 cm Coaxial Programmable Automatic Biopsy System (Coaxial Achieve^®^, CareFusion Corporation, San Diego, CA, USA) (Figure 2). The punch is a fully automated system with a 20 mm sample collection area and a thin-walled cannula for tissue samples as large as possible. The punching cylinder measures up to 18.0 mm × 1.3 mm with round diameter and was preserved with 4% formalin on site according to the standard of the Institute of Pathology. Due to the total length of 11 cm of the lance of the system, the biopsy was also successful in patients with soft tissue accumulations in the hip or knee region (body mass index (BMI) > 30) or patients with, e.g., anasarca, oedema or other underlying diseases.

### 2.3. Test Regimes

All patients had undergone ambulatory preoperative diagnosis for microbiology as well as for histopathology according to the inclusion criteria. In addition, microbiological and histopathological samples were collected and analysed intraoperatively in all patients. In the ambulatory setup one microbiological and one histopathological sample was taken. According to the intraoperative in-house-standard microbiological samples from 4–5 defined locations were taken. At least one histological sample from the PJI suspected area was taken. More histopathological samples were collected, if more sites showed suspicion of infection.

In order to determine the sensitivity and specificity of the preoperative test regimes the following three comparisons were performed: Test Regime I: preoperative microbiology only vs intraoperative diagnosis; Test Regime II: preoperative histopathology only vs intraoperative diagnosis; Test Regime III: combination of preoperative microbiology and histopathology vs intraoperative diagnosis.

In order to perform the microbiological and histopathological testing procedures, the samples were sent respectively for microbiological and histopathological analyses to the Institute of Microbiology, Virology and Hygiene and the Institute of Pathology of the University Rostock Medical Center. Microbiological diagnostics were carried out according to German microbiological standards [17,18] at the national accreditation organisation of the Federal Republic of Germany (DAkkS) DIN EN ISO 15189 and DIN EN ISO/IEC 17025 accredited microbiological laboratory. As a criterion for the microbiological findings of the puncture, the detection of a pathogen from the joint was considered positive. For the intraoperative microbiological findings, a result with the number “ample” or two positive pathogen detections was considered as infection. Analysis of the prosthetic neosynovialis in punch biopsy was performed following the consensus classification according to Morawietz/Krenn [12]. Counting of neutrophilic granulocytes in ten visual fields > 10 granulocytes/high power field (HPF) findings evaluated as positive. The histopathological and microbiological samples were compared and evaluated.

### 2.4. Statistics

The results were collected using Microsoft Excel 2016 (Microsoft, Redmond, WA, USA) and evaluated via IBM SPSS Statistics 25 (IBM Corp., New York, USA). Descriptive statistics were calculated for continuous and categorical variables. Cross tables were generated, and a Chi square test, tests for normal distribution and a Mann–Whitney U Test were performed in SPSS. *p*-values < 0.05 were considered as statistically significant. The measures of diagnostic accuracies was performed with “Tool: Measures of diagnostic accuracies of a 2 × 2 table” for Microsoft Excel 2016 (Dr. Thomas Keller, ACOMED statistik, Fockestr. 57, 04275 Leipzig, Germany). If not stated otherwise, all data is presented as me ± standard deviation (SD) [median; Min-Max].

### 2.5. Cost Analysis

An analysis of the costs of the proceedings in Germany was carried out; this may vary for other countries.

### 2.6. Ethics Vote and Data Privacy

Ethics approval for the study was granted by the local ethics committee (registration number A2018-0048), data protection requirements were observed, consent to participate was not necessary.

## 3. Results

### 3.1. General Patient Data and Surgical Method

A total number of 102 patients fulfilled the inclusion criteria. In detail, participants were 52.0% men (*n* = 53) and 48.0% women (*n* = 49), 8.8% quoted regular tobacco and 10.8% regular alcohol consumption. The average age was 71.03 ± 10.69 (74.5; 30–87) years, the average BMI was 30.4 ± 5.76 (29.07; 19.0–54.0) kg/m^2^. The American Society of Anesthesiologists score (ASA) showed 4.9% patients with ASA 1, 37.3% patients with ASA2, 49% patients with ASA 3, 4.9% patients with ASA 4 [19]. While 43.1% patients showed pain in the joint at rest and 27.5% pain under load, 3.9% of the patients reported no pain. In 25.5% of the cases pain was not documented. A total of 55.1% hip arthroplasties and 44.9% knee arthroplasties were examined, of which 74.5% were primary and 25.5% revision endoprostheses, 53.9% of the prostheses were cemented, 38.2% non-cemented.

The average intervention time of the punch and microbiological puncture was 9 min and was carried out under local anesthesia.

### 3.2. Preoperative Diagnosis

The preoperative microbiology was positive in 15.7% (16) of the cases, the preoperative histopathology revealed positive results in 13.7% (14) of the cases. In one case synovial fluid could not been drawn, a punch biopsy was obtained for evaluation, this showed no evidence of pathogens. In the histopathological evaluations of the 102 patients, the analysis according to Morawietz/Krenn showed 20 cases of wear particle induced type (Type I), 13 cases for infectious type (Type II), 1 case for combined type (Type III), and 2 cases for indeterminate type (Type IV), respectively. The majority of 66 punch biopsies could not be clearly identified according to Morawietz/Krenn. The histopathology corresponded most closely to Type IV but did not exactly match the description and had no clinical relevance for PJI due to the absence of signs of infection in histopathology.

### 3.3. Intraoperative Diagnosis

#### 3.3.1. Microbiology

Of the 102 screened patients suspected to suffer from a periprosthetic infection 27 (26.5%) cases were confirmed intraoperatively. Two patients who were positive in the preoperative microbiology testing did not show signs of infection in the intraoperative samples. In 13 (12.7%) patients the preoperative microbiology was negative but showed positive microbiological results by intraoperative sampling. In these cases, the pathogens were located mainly in closed compartments (e.g., behind the cup or stem) without directly corresponding to the joint. In those patients with a negative preoperative sample but a positive intraoperative sample, microbes were detected mainly at localisations such as the capsule with 62.5%, the cup with 85.7% and the tibia 100%, but less so in the incision with 25.0% or the femur with 28.6%. In patients where the positive intraoperative sample was matched by positive preoperative microbiology testing, which occurred in 13.7% of cases (14 patients), the infection was more widespread and pathogens were located with 62.5% in the incision, with 87.5% in the capsule, with 83.3% in the cup, with 75.0% in the femur and with 50% in the tibia. Table 1 gives an overview of the microbial spectrum detected in the arthrocentesis (pre-operative) and intraoperative microbial samples.

#### 3.3.2. Histopathology

**Figure 3:**
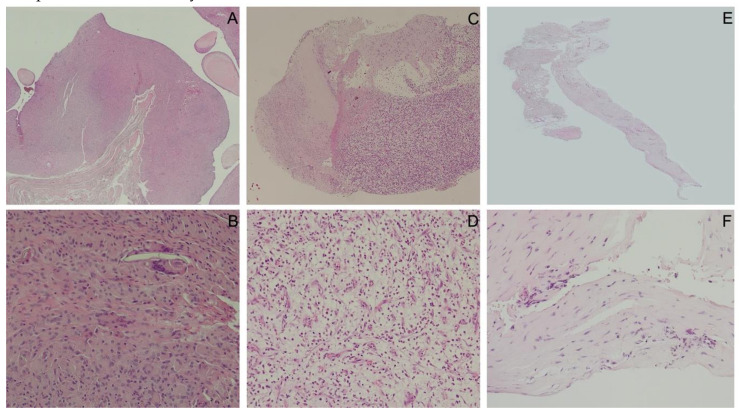
Histopathological specimens of Morawietz/Krenn Type I ((**A**): scanning magnification view of 4-fold and (**B**): 20-fold magnification); Type II ((**C**): scanning magnification view of 4-fold and (**D**): 20-fold magnification); Type IV ((**E**): scanning magnification view of 4-fold and (**F**): 20-fold magnification).

In the intraoperative histopathological evaluations, the analysis according to Morawietz/Krenn showed 51 cases of wear particle induced type (Type I), 10 cases for infectious type (Type II), 7 case for combined type (Type III), and 13 cases for indeterminate type (Type IV), respectively. A total of 17 biopsies could not be clearly identified according to Morawietz/Krenn. Those corresponded most closely to Type IV but did not exactly match the description (Figure 3). In 4 cases, the histological sample could not be analyzed.

#### 3.3.3. Analysis of the Sensitivity and the Specificity of the Different Test Regimes

Of the 27 intraoperatively confirmed cases of PJI preoperative testing resulted in 9 cases testing positive in both, microbiological and histopathological analyses, 5 cases displayed positive microbiological results while the histopathology was negative, whereas a further 5 cases showed positive histopathology results but no detection of pathogens. In 8 cases of confirmed PJI preoperative testing was unable to detect the infection (Figure S1).

Based on these results the following sensitivities and specificities were calculated:

Test Regime I (preoperative microbiology only vs. intraoperative diagnosis): In 51.9% of the PJI cases there was a preoperative as well as an intraoperative detection of infection in the microbiological specimen (Table 2). The test specific sensitivity was 51.9% (95% width of CI: 31.9% –71.3%) and the specificity was 97.3% (95% width of CI: 90.7%–99.7%).

Test Regime II (preoperative histopathology only vs. intraoperative diagnosis): In 51.9% of the PJI cases there was a preoperative as well as an intraoperative detection of infection in the histopathological specimen (Table 2). The test specific sensitivity was 51.9% (95% width of CI: 31.9%–71.3%) and the specificity was 100.0% (95% width of CI: 95.2%–100.0%).

Test Regime III (combination of preoperative microbiology and histopathology vs. intraoperative diagnosis): In 70.4% of the confirmed PJI cases, there was a preoperative as well as an intraoperative detection of infection in the microbiological and histopathological specimen. The combination of the two tests resulted in a sensitivity of 70.4% (95% width of CI: 46.0%–83.5%) and a specificity of 97.3% (95% width of CI: 90.7%–99.7%). Statistically there was no significant difference in sensitivity between the 3 diagnosis groups (*p* = 0.282). However, the combination of both preoperative diagnostical procedures increased the sensitivity to detect PJI by 18.5% (Table 2). Five of the 27 septic cases would not have been detected as infection preoperatively when relying on one test result only, i.e., without the combination of preoperative microbiology and histopathological punch biopsies.

### 3.4. Complications of the Intervention

A total of 102 patients received arthrocentesis of a hip or knee prosthesis in the specified period. The only complication observed during this period was one intraarticular bleeding of a knee joint in one patient due to the double anticoagulation therapy the patient was taking. No further complications were recorded. The prescription of non-steroidal anti-inflammatory drugs (NSAIDs) after the intervention was not necessary unless there was already pain before.

### 3.5. Cost Analysis

The total costs for the minimally invasive procedure for histopathological specimen collection, including material costs, medical costs and histopathological evaluation, were a maximum of 75 €. The total time required was on average 9 ± 1.31 (8.17; 7–14) minutes.

## 4. Discussion

The results of the present study are of high clinical relevance. They represent a clear gain in diagnostic certainty due to the clear increase in sensitivity of over 18.0% with regard to the diagnosis of periprosthetic infection. On their own, histopathology and microbiology showed similar sensitivities in detecting periprosthetic infections. In combination (Test Regime III), however, the sensitivity increased considerably with little effort and without major risks for the patient to detect periprosthetic infections. The extraction of the neosynovia was performed in an ambulatory setting, in a short period of time at low cost and with low risk for the patient. The procedure itself was safe and easy to learn after a short training period.

It must also be mentioned that in addition to the diagnosis of PJI, documentation plays an important role and the information chain with all participants must be maintained accordingly. This is especially important when antibiotics are used, as well as the communication of the further treatment concept.

### 4.1. Microbial Spectrum

The analysed microbial spectrum showed that the sensitivity of intraoperative samples is higher for some pathogens. A possible cause may be the lower number of samples in arthrocentesis (*n* = 1) in contrast to the intraoperative number of samples (*n* = 4–5). To increase the sensitivity of arthrocentesis (microbiologically), a punch could be obtained for further microbiological examination from the neosynovia. However, it should be noted that multiple sample taking can lead to a higher risk of infection during arthrocentesis, especially as the advantage of additional samples is questionable since we found the intraoperative pathogens often in difficult-to-access places or closed compartments as described. The relatively high proportion of pathogens of the skin flora in the arthrocentesis, which does not correspond to the intraoperative findings, can be an indication of contamination of the ambulatory sample, despite skin incision and thorough disinfection. Further handling of the sample in the ambulatory setup could also be a possible cause of contamination. On the other hand, intraoperative rinsing procedures might result in less detection of skin pathogens.

### 4.2. Testing of the Synovial Fluid

A substantial part of the minor criteria is devoted to the investigation of synovial fluid in addition to microbiology. The white blood cell count (WBC) and positive detection of alpha-defensin, an antimicrobial peptide (AMP) that has been evaluated for synovial PJI detection [20], have a high significance. However, the results must be interpreted with caution, as the type of pathogen can influence the threshold values for serum and synovial markers [21]. In this study, the sensitivity of the exclusive microbiological examination was 51.85% and the specificity was 97.33%. The literature indicates therefore, that any examination of synovial fluid should be accompanied by further biomarker testing in addition to the microbiological culture [22].

The increasing availability of multiplex-PCR assays, that allow for the rapid detection of pathogens with information on their resistance patterns in a timely manner, enables further diagnosis of the synovial fluid [23]. These are currently only available at large centres and can only show their diagnostic and consecutively therapeutic potency if there is a close (spatial) connection between the operational unit and the microbiological department [24]. In summary, these examinations can serve as a further element in infection diagnosis, but do not lead to absolute diagnostic certainty [9,25].

Pathogen negative preoperative punctures of the joints poses a challenge. In the present study it could be shown that the sensitivity of the microbiological examination of synovial fluid is only about 51%. It was further shown, that if this examination was positive, the infection had usually spread throughout the entire joint. In culture-negative preoperative punctures, however, in addition to the bacteria-free nature of the joint, there is the possibility that bacteria may be present in the bone socket of the prosthesis without contact to the joint. Intraoperatively, with a high probability of over 85.0% infection was found behind the cup of the prosthesis or with 62.5% in the joint capsule without being detected at further intra-articular infection sites when pre-operative microbiological detection failed, i.e., when there was rather a low-grade infection. This was similarly described in the literature, therefore the recommendation was put forward that at least 4 or 5 microbiological samples from several spots should be taken intraoperatively [5,26].

### 4.3. Analysis and Value of Histopathology

According to the guidelines of Morawietz et. al, in this study a periprosthetic membrane was evaluated as PJI positive if > 10 granulocytes/high power field (HPF) are found. In the literature, there are indications that a cut-off value of 23 neutrophils in 10 high power fields is more appropriate for diagnosis of PJI [12,27]. In the minor criteria of MSIS the histopathological sample is not considered, whereas the consensus criteria, with 3 points, give histopathology a high priority among the minor criteria [6,28]. One possibility why MSIS does not take this into consideration may be the lack of a sufficient minimally invasive biopsy collection. Arthroscopic sampling is a possibility for histopathological sample acquisition but it is an invasive procedure with additional risks for the patient, such as nausea and vomiting, circulation dysregulation, scratching of the endoprosthesis surface, wound healing disorders, thromboembolism, bleeding into the joint or infection. However, more information can be obtained here (mechanical problems, more targeted sample collection under view), but according to Classen et al., minimally invasive diagnostic tests should be used in advance [14].

With this study we can confirm the high value of histopathology in the ICM criteria because of the same sensitivity and approximately the same specificity compared to the microbiological synovial sample. In addition, the combined use of both methods can achieve a clear increase in sensitivity by more than 18.0% and thus provides significantly more reliability also for diagnosis of low-grade infection. According to the results of this study, a preoperatively gained histopathology sample should always be interpreted together with another synovial biomarker and a microbiological culture, WBC should be included in every synovial sample. It should also be noted that without the punch biopsy, 5 of 27 (18.5%) septic cases would not have been detected as infection preoperatively. Furthermore, the histopathological sample can provide information about the activity of the inflammation in the joint, as differentiation between florid and chronic infection is possible. It should be noted that in 66 cases the joint capsule was not accurately hit. Therefore, the sampling error is the major disadvantage of the punching technique. In this case, a significant increase in accuracy could be achieved with new punches or techniques and continuous training of the surgeons. Nevertheless, this study could prove the high clinical relevance of preoperative histopathology. If the right spot is hit, the periprosthetic membrane is the best place to diagnose PJI [11]. Another disadvantage of the histopathological detection method for periprosthetic infections, however, is the lack of detection of fungal infections of the joint. In case of suspicion, fungal infection can only be detected with special staining methods but not in the native specimen. The combination of histopathology and microbiology has a great advantage here.

### 4.4. Cost and Benefit Analysis

For diagnostic purposes, an arthroscopic intervention for intraoperative specimen collection can be performed, as mentioned above. Sample size and location can be determined more easily here. In comparison, however, the use of a punching system for the minimally invasive collection of neosynovia is significantly more cost- and time-effective. Furthermore, the complication rate for the correct use of the punch is very low overall and thus clearly speaks in favour of the minimally invasive punch intervention.

## 5. Conclusions

The diagnosis of PJI is complex and a single method is currently not able to reliably detect an infection due to its inherent limitations in sensitivity and specificity. The combination of several methods may circumvent these issues. Additional preoperative histopathological sampling can lead to an increase in sensitivity if combined with other methods. The significance of histopathology should be considered as high value and as a major criterium of PJI. Furthermore, histopathology can differentiate between florid and chronic infection and provide indications of aseptic loosening reasons.

With the current technique the extraction of the histological specimens can be performed in an ambulatory setting within a short period of time at low cost and with low risk for the patient while simultaneously increasing the diagnostic certainty. However, further investigations should concentrate on the development of newer biopsy systems to allow collection of synovial fluid and histopathological specimen with one apparatus.

## Figures and Tables

**Table 1:** Descriptive Overview of Microbial Spectrum.

Pathogen	Microbial Spectrum (%, (*n*))	
	Arthrocentesis	Intraoperative
Gram-positive bacteria		
Aerobic		
* Staphylococcus epidermidis*	33.33 (6)	32.14 (9)
* Staphylococcus haemolyticus*	0.00 (0)	7.14 (2)
* Staphylococcus capitis*	0.00 (0)	3.57 (1)
* Staphylococcus lugdunensis*	5.56 (1)	3.57 (1)
* Staphylococcus aureus*	5.56 (1)	0.00 (0)
* Staphylococcus hominis*	5.56 (1)	0.00 (0)
* Staphylococcus saphrophyticus*	5.56 (1)	0.00 (0)
Coagulase negative staphylococci	5.56 (1)	0.00 (0)
* Streptococcus anginosus*	5.56 (1)	3.57 (1)
* Streptococcus parasanguinis*	0.00 (0)	3.57 (1)
* Streptococcus gordonii*	5.56 (1)	0.00 (0)
* Enterococcus faecalis*	5.56 (1)	10.71 (3)
Anaerobic		
* Actinomyces odontolyticus*	0.00 (0)	3.57 (1)
* Finegoldia magna*	0.00 (0)	3.57 (1)
* Cutibacterium acnes*	0.00 (0)	7.14 (2)
* Cutibacterium avidum*	5.56 (1)	0.00 (0)
Cutibacterium (formerly Propioni) species	0.00 (0)	3.57 (1)
Gram-negative bacteria		
Aerobic		
* Escherichia coli*	11.11 (2)	7.14 (2)
Yeast		
*Candida parapsilosis*	0.00 (0)	3.57 (1)
*Candida albicans*	5.56 (1)	3.57 (1)
*Candida glabrata*	0.00 (0)	3.57 (1)

**Table 2:** Test Regime for Arthrocentesis Compared to Intraoperative Results.

Test Regime			Intraoperative		Results
			Negative	Positive	
I	Preoperative: Microbiology	negative	73 (97.3%)	13 (48.1%)	Sensitivity 51.9% (95%-width of CI: 31.95%–71.33%) Specificity 97.3% (95%-width of CI: 90.7%–99.68%)
		positive	2 (2.7%)	14 (51.9%)	
		total	75	27	
II	Preoperative: Histopathology	negative	75 (100%)	13 (48.1%)	Sensitivity 51.9% (95%-width of CI: 31.95%–71.33%) Specificity 100.0% (95%-width of CI: 95.2%–100.0%)
		positive	0 (0%)	14 (51.9%)	
		total	75	27	
III	Preoperative: Combination	negative	73 (97.3%)	8 (29.6%)	Sensitivity 70.4% (95%-width of CI: 46.04%–83.48%) Specificity 97.3% (95%-width of CI: 90.7%–99.68%)
		positive	2 (2.7%)	19 (70.4%)	
		total	75	27	

## Data Availability

The data were collected and evaluated within the Orthopaedic Clinic and Policlinic, University Rostock Medical Center, Rostock, Germany. The collected data obtained have been stored and are available at Orthopaedic Clinic and Policlinic.

## Supplementary Materials

The following are available online at https://www.mdpi.com/2077-0383/9/10/3364/s1. Figure S1: Proportions of positive preoperative test results in intraoperative confirmed PJI cases.
