# Supplementary figures and images for: Increased Diagnostic Certainty of Periprosthetic Joint Infections by Combining Microbiological Results with Histopathological Samples Gained via a Minimally Invasive Punching Technique
Source: J Clin Med. 2020 Oct 20;9(10):3364. doi: 10.3390/jcm9103364 (PMC7594052; doi:10.3390/jcm9103364)

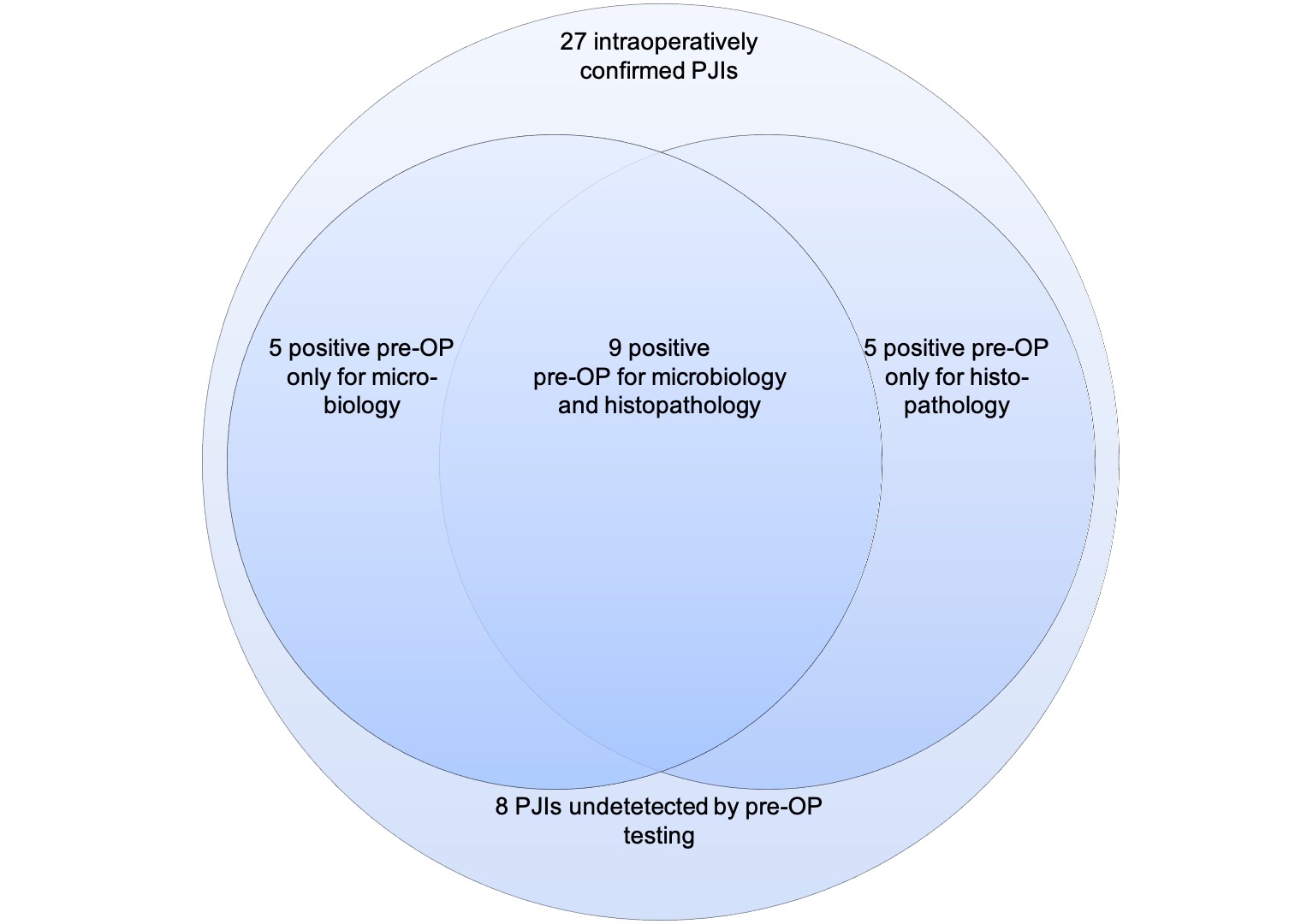

Supplement: Supplementary file 1 [file jcm-09-03364-s001.jpg]
